# Missed opportunities in full immunization coverage: findings from low- and lower-middle-income countries

**DOI:** 10.3402/gha.v9.30963

**Published:** 2016-05-03

**Authors:** María Clara Restrepo-Méndez, Aluísio J. D. Barros, Kerry L. M. Wong, Hope L. Johnson, George Pariyo, Fernando C. Wehrmeister, Cesar G. Victora

**Affiliations:** 1 International Center for Equity in Health, Federal University of Pelotas, Pelotas, Brazil; 2 International Center for Equity in Health, Postgraduate Program in Epidemiology, Federal University of Pelotas, Pelotas, Brazil; 3 Gavi, The Vaccine Alliance, Geneva, Switzerland; 4 Johns Hopkins University, Bloomberg School of Public Health, Baltimore, MD, USA

**Keywords:** vaccines, vaccination, immunization, child health, health services

## Abstract

**Background:**

An estimated 23 million infants are still not being benefitted from routine immunization services. We assessed how many children failed to be fully immunized even though they or their mothers were in contact with health services to receive other interventions.

**Design:**

Fourteen countries with Demographic and Health Surveys and Multiple Indicator Cluster Surveys carried out after 2000 and with coverage for DPT (Diphtheria-tetanus-pertussis) vaccine below 70% were selected. We defined full immunization coverage (FIC) as having received one dose of BCG (bacille Calmette-Guérin), one dose of measles, three doses of polio, and three doses of DPT vaccines. We tabulated FIC against: antenatal care (ANC), skilled birth attendance (SBA), postnatal care for the mother (PNC), vitamin A supplementation (VitA) for the child, and sleeping under an insecticide-treated bed-net (ITN). Missed opportunities were defined as the percentage of children who failed to be fully immunized among those receiving one or more other interventions.

**Results:**

Children who received other health interventions were also more likely to be fully immunized. In nearly all countries, FIC was lowest among children born to mothers who failed to attend ANC, and highest when the mother had four or more ANC visits Côte d'Ivoire presented the largest difference in FIC: 54 percentage points (pp) between having four or more ANC visits and lack of ANC. SBA was also related with higher FIC. For instance, the coverage in children without SBA was 36 pp lower than for those with SBA in Nigeria. The largest absolute difference on FIC in relation to PNC was observed for Ethiopia: 31 pp between those without and with PNC. FIC was also positively related with having received VitA. The largest absolute difference was observed in DR Congo: 41 pp. The differences in FIC among whether or not children slept under ITN were much smaller than for other interventions. Haiti presented the largest absolute difference: 16 pp.

**Conclusions:**

Our results show the need to develop and implement strategies to vaccinate all children who contact health services in order to receive other interventions.

## Introduction

Immunization is perhaps one of the most successful and cost-effective public health interventions for children, which has helped to drive the reduction in child mortality worldwide. The number of deaths caused by traditional vaccine-preventable diseases such as measles, neonatal tetanus, and pertussis has fallen from an estimated 705,487 in 2000 to 165,770 in 2015 (1–3). Coverage of vaccines has been substantially expanded over the past decades since the inception of the Expanded Programme on Immunization and also the introduction of new vaccines (2). Despite such progress, 23 million children still failed to receive the basic set of routine immunizations scheduled for their first year of life in 2012 (2–4).

The Global Vaccine Action Plan (GVAP) is a strategy endorsed in 2012 by the 194 Member States of the World Health Assembly to ensure equitable access to existing vaccines (2). Reaching unvaccinated children – typically in poorly-served remote rural areas, deprived urban settings, fragile states, and strife-torn regions – is essential if the goals of the GVAP are to be met (2).

Several studies have examined the influence of demographic and socio-economic factors on disparities in child immunization in underprivileged settings (5–9). However, less is known on how health-systems related factors affect such disparities. We used extensive data available from nationally representative surveys to assess levels of full immunization coverage (FIC) in relation to coverage of other health interventions, thus allowing the identification of children who failed to be fully immunized even though they and their mothers were in contact with health services to receive other interventions.

## Methods

Our data sources included Demographic and Health Surveys (DHS) (10) and Multiple Indicator Cluster Surveys (MICS) (11). Seventeen countries were selected according to *a priori* criteria, including coverage below 70% with three doses of DPT vaccine and representation of different world regions. We based our selection on DPT as the coverage with three doses of DTP is a standard indicator of the reach of national immunization programs. Data on DPT coverage was based on World Health Organization (WHO) and United Nations Children's Emergency Fund (UNICEF) estimates from 2013 (3). The countries included were Afghanistan, Cameroon, Democratic Republic of the Congo (DR Congo), Ethiopia, Uganda, Central African Republic (CAR), Chad, Côte d'Ivoire, Guinea, Haiti, Liberia, Mauritania, Nigeria, Somalia, Timor-Leste, Papua New Guinea, and South Sudan. For each selected country, we used the last available survey carried out after 2000. Surveys were available for 14 out of the 17 target countries (nine DHS and five MICS). Three countries did not have available data (Afghanistan, Papua New Guinea, and South Sudan).

The outcome under study was FIC, defined as the proportion of children aged 12 to 23 months at the time of the survey who had received one dose of BCG, three doses of DPT (or other polyvalent vaccine including DPT), three doses of polio vaccine, and one dose of measles vaccines (either as monovalent vaccine or as measles-containing vaccine combinations with other immunogens). In all the studied countries, the administration of the vaccines of interest was scheduled for the first year of life. The information on administered vaccines was based on the record of the vaccination extracted from vaccination cards and the mother's report of the vaccination for children with either no card or no record of the vaccination. We also estimated missed opportunities, which were defined as 100% minus FIC, that is, the percentage of children who failed to be fully immunized in a particular group.

FIC was stratified according to the following variables: antenatal care (ANC; categorized as zero, one to three, and four or more visits), skilled birth attendance (SBA), postnatal care for the mother within 2 days of giving birth (PNC), vitamin A supplementation for the child in the 6 months previous to the survey (VitA), the child slept under an insecticide-treated bed-net (ITN) on the night before the survey, and the child had ever received a health/vaccination card (HVC).

For MICS surveys, no information was available on PNC; and for surveys carried out before 2009, the number of ANC visits was not available so that the ANC variable was coded as any versus none. Supplementary File 1 presents the source of data for each country and the availability of intervention coverage variables for the analyses.

In MICS surveys, information on ANC and SBA and child vaccination are stored in different datasets, and it was necessary to link them. Given that some children included in the survey do not have mothers living in the same household, information on ANC and SBA was missing for 3 to 6% of the children.

### Statistical analyses

We used Poisson regression with robust variance (12) to assess the association between FIC and each health intervention. Survey sample designs were taken into account when estimating the coverage of full immunization for each country using the *svy* set commands available in Stata. When the unweighted number of observations in a specific subgroup was fewer than 25, results were omitted. Analyses were carried out in Stata^®^/MP13 (StataCorp LP, College Station, Texas, United States).

All analyses were based on publicly available data from national surveys. Ethical clearance was the responsibility of the institutions that administered the surveys.

## Results

Table 1 presents the unweighted numbers of children (total and fully immunized) for each country included in the analyses. FIC is below 60% in all countries, varying from 11% in Chad to 54% in Cameroon. Coverage with each separate vaccine is also shown in Supplementary File 2. In general, the coverage of DPT tends to be lower than the other vaccines (measles, BCG and polio) in most of countries.

**Table 1:** Sample sizes and FIC by country

UN region	Country	Sample size (children)	*N* (fully immunized)	FIC* (%)
East Asia & Pacific	Timor-Leste	1805	988	52.6
Eastern & Southern Africa	Uganda	1427	769	52.5
Eastern & Southern Africa	Ethiopia	1927	565	24.6
Eastern & Southern Africa	Somalia	1096	113	11.6
Latin America & Caribbean	Haiti	1370	640	45.8
West & Central Africa	Cameroon	2286	1241	53.6
West & Central Africa	Côte d'Ivoire	1417	716	50.5
West & Central Africa	DR Congo	2318	1122	49.8
West & Central Africa	Liberia	996	352	39.1
West & Central Africa	Guinea	1115	430	37.4
West & Central Africa	Mauritania	1677	651	35.3
West & Central Africa	Nigeria	4755	1458	33.2
West & Central Africa	CAR	2015	324	17.3
West & Central Africa	Chad	901	145	11.4

* FIC was calculated taking into account sampling weights.

**Fig. 1:**
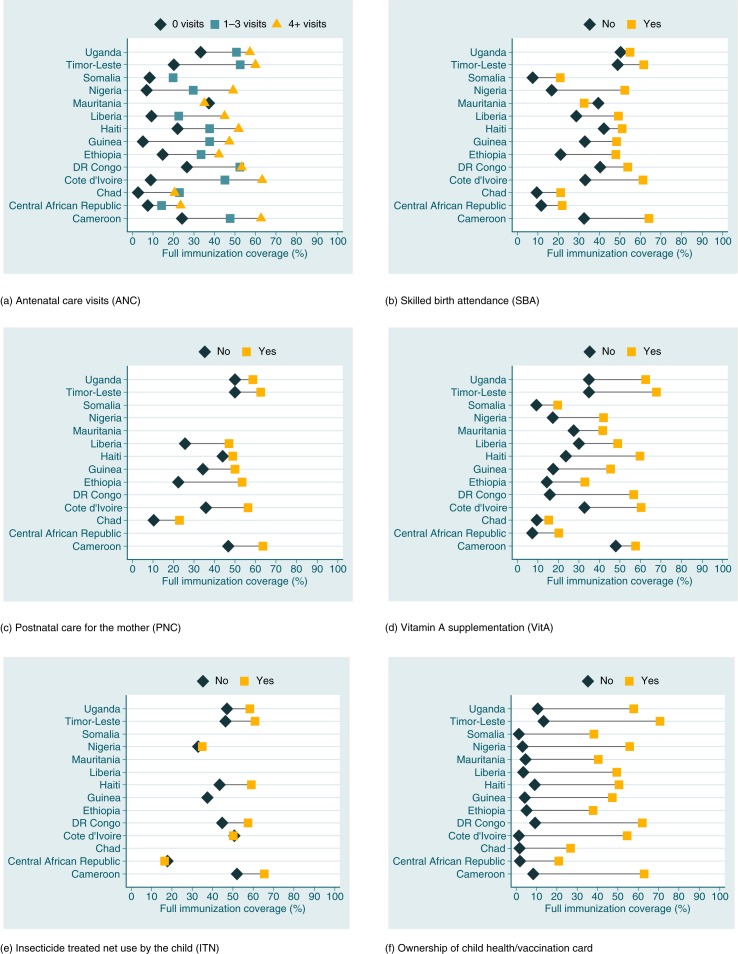
Full immunization coverage (%) according to other health care interventions. (a) Full immunization coverage (%) according to number of antenatal care visits. The horizontal lines link the extreme ANC categories. Mauritania: Information on ANC limited to having at least one visit during pregnancy. Red circle for no coverage and blue circle for at least one. Somalia did not have sufficient number in the category with ANC four times or more. (b) Full immunization coverage (%) according to skilled birth attendance. (c) Full immunization coverage (%) according to postnatal care for the mother. (d) Full immunization coverage (%) according to vitamin A supplementation. (e) Full immunization coverage (%) according to insecticide-treated net use by the child. Guinea did not have sufficient number in the category with no insecticide-treated bed-net. (f) Full immunization coverage (%) according to ownership of child health/vaccination card. Note: Lines are blank when data are not available.

Comparisons among countries for each intervention are shown in Fig. 1. FIC estimates, prevalence ratios and their 95% confidence intervals (95% CI) are presented in Supplementary File 3. Large differences in FIC were observed according to coverage with other health interventions in the 14 surveys analyzed. In nearly all countries with information on the number of ANC visits, FIC was lowest among children born to mothers who failed to attend ANC, and highest when the mother had four or more visits. In Somalia, the number of mothers with four or more visits was very small, and for Mauritania, only information on at least one ANC visit was available. In absolute terms, Côte d'Ivoire presented the largest difference in coverage: 54.4 percentage points between ANC of four or more and lack of ANC.

All countries had information on SBA, and it was associated with higher FIC coverage at the 0.05 P level. The differences were not as large as for ANC, but still FIC among children without SBA was 35.6 percentage points lower than for those with SBA in Nigeria, the country with the largest gap.

Information on PNC was available for nine countries, in all of which care was related with higher FIC. The largest absolute difference was observed for Ethiopia: 30.9 percentage points between those without and with postnatal care.

Data on VitA were available for all countries, in all of which FIC was positively related with having received VitA. The largest absolute difference was observed in DR Congo: 40.7 percentage points.

Finally, nine countries had information on whether or not children slept under ITN the previous night. The differences were generally much smaller than for other interventions. This may be related to the fact that ITNs may be obtained through different channels than the previous interventions, which are provided through the health system. For CAR, Côte d'Ivoire and Nigéria, there was a very small difference between groups. Haiti presented the largest absolute difference, 15.6 percentage points.

**Fig. 2:**
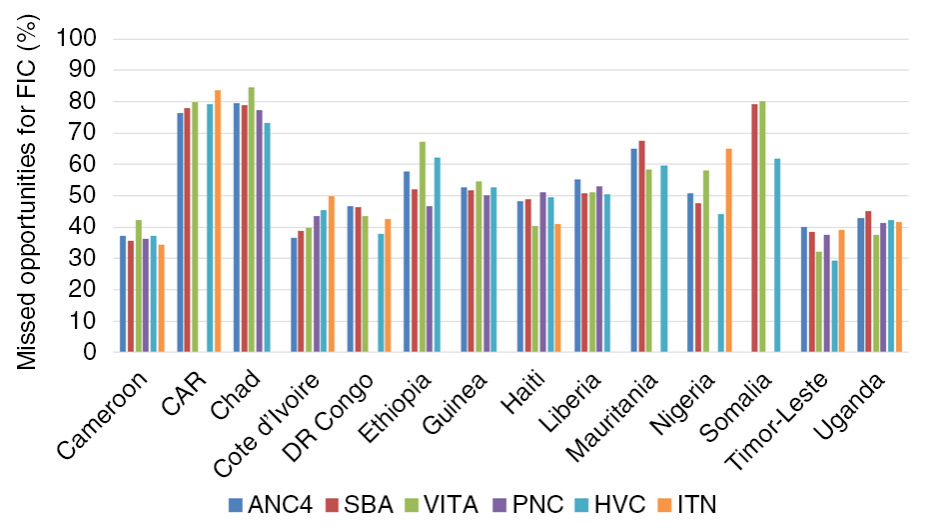
Missed opportunities analyses: percent of children who were not fully immunized in spite of being in contact with the health system to receive other interventions. ANC4: antenatal care (four or more visits); SBA: skilled birth attendant; VitA: Vitamin A supplementation for the child in the 6 months previous to the survey; PNC: Postnatal care for the mother; HVC: Ownership of child health/vaccination card; ITN: Sleeping under an insecticide-treated bed-net on the night before the survey. Note: blank bars represent lack of information on a particular variable or sample size below 25.

We also estimated the percentage of children who failed to achieve FIC status, even though they or their mothers were in contact with health services (Fig. 2). The percent of missed opportunities, meaning the percentage of mothers and children who received one or more of the five health interventions as recommended, yet failed to have their children fully vaccinated, was 50% or greater in 8 out of 14 countries (CAR, Chad, Ethiopia, Guinea, Liberia, Mauritania, Nigéria, and Somalia).

## Discussion

Our results document the presence of co-coverage (13) between FIC and the other interventions studied, meaning that children who were fully vaccinated were also more likely to have received the other interventions studied. This is probably related to several factors, including greater geographical access to health services (e.g. living in urban areas, or living close to a facility for rural families). Cultural and socio-economic factors may also play a role. Some families are more aware of the benefits of health interventions (e.g. parents who are more educated) and can also afford the direct and indirect costs of accessing the services if these interventions are delivered (e.g. families with higher socio-economic position). Of all subgroups studied, FIC was particularly high among children whose mothers had four or more ANC visits. Similarly to FIC, ANC requires repeated visits to health providers and is more likely to be used by mothers to whom services are more easily accessible.

Analyses of why missed opportunity patterns varied among the countries studied are beyond the scope of the present article. These differences are probably related to the strategies used to deliver each intervention, as some of them are delivered at the health facility level, community level, or through campaigns. For example, countries where vitamin A or ITNs are distributed through campaigns that also provide vaccinations would be expected to lead to a higher magnitude of coverage of immunization among these interventions. Testing this hypothesis, however, will require additional data on delivery approaches to these interventions in each country.

Although coverage among different types of intervention tends to be clustered at the mother and child level, there were many missed opportunities for vaccination. In all countries studied, there were frequent failures for full immunization among those who contacted with health services in order to receive other maternal and child health interventions. We found similar results when we analyzed each individual vaccine according to coverage with health interventions. Nevertheless, we observed that the coverage of vaccines that require multiple doses (DPT and polio) was lower than the coverage of vaccines that require single doses (measles and BCG) in most countries.

Our analyses have limitations. The tabulations of health and vaccination cards need to be interpreted with care, since the vaccines recorded for a child without a card will be based only on the mother's recall, and subject to recall and information bias. In this case, underreporting is to be expected. Coverage data being reported here are solely based on survey information. International agencies estimate coverage using a combination of data from surveys and from health information systems, and therefore the coverage levels reported here will not necessarily be consistent with those reported in United Nations documents. However, FIC cannot be calculated on a routine basis because this indicator combines four different vaccines at the level of each individual child.

A comparison of results among different countries should be made with caution. For example, unlike a comparison of gaps according to wealth quintiles, which have similar relative sizes in all countries, comparisons of FIC gaps between children (and mothers) who received or failed to receive a given intervention are affected by the relative sizes of these groups. For this reason, we did not attempt to aggregate the results of our analyses across the 14 countries.

Our results suggest that, in spite of global progress in immunization, substantial proportions of children in many countries still fail to benefit from all basic vaccines. Several challenges must be tackled in order to achieve the GVAP goal of delivering universal access to immunization (2). These include the need to explore additional cross-programmatic and cross-sectoral strategies to avoid missed opportunities for vaccinating children who contact health services for other interventions, particularly in low and middle income countries. In addition, further research is required to understand the political, social, economic, and health-related factors that account for missed opportunities in vaccinations. We hope that these findings will inform policy debates on strategies to reduce missed opportunities in vaccination, as well as showcase the importance of taking into account other programs when tracking progress in immunization coverage.

## Supplementary Material

Missed opportunities in full immunization coverage: findings from low- and lower-middle-income countries

## Paper context

Despite global progress in immunization, 23 million children still fail to receive the basic set of routine vaccines. We used data from national surveys to assess levels of FIC in relation to coverage of other health interventions, thus allowing identification of children who failed to become fully immunized when they and their mothers were in contact with health services to receive other interventions. Our results suggest the need to develop and implement strategies for reaching children who contact health services for other interventions to avoid missed opportunities for vaccinating children.
