# Supplementary material for: Missed opportunities in full immunization coverage: findings from low- and lower-middle-income countries
Source: Glob Health Action. 2016 May 3;9:10.3402/gha.v9.30963. doi: 10.3402/gha.v9.30963 (PMC4856841; doi:10.3402/gha.v9.30963)
Supplement: Missed opportunities in full immunization coverage: findings from low- and lower-middle-income countries [file GHA-9-30963-s001.pdf]

Web-appendix 1. Data sources used in the analyses, showing stratification variables available in each survey

Country	Survey year	Source	ANC	SBA	VitA	PNC	HVC	ITN
1. Cameroon	2011	DHS	✓	✓	✓	✓	✓	✓
2. CAR	2010	MICS	✓	✓	✓	✗	✓	✓
3. Chad	2004	DHS	✓	✓	✓	✓	✓	✗
4. Côte d'Ivoire	2011	DHS	✓	✓	✓	✓	✓	✓
5. DR Congo	2010	MICS	✓	✓	✓	✗	✓	✓
6. Ethiopia	2011	DHS	✓	✓	✓	✓	✓	✗
7. Guinea	2005	DHS	✓	✓	✓	✓	✓	✓
8. Haiti	2012	DHS	✓	✓	✓	✓	✓	✓
9. Liberia	2007	DHS	✓	✓	✓	✓	✓	✗
10. Mauritania	2007	MICS	*	✓	✓	✗	✓	✗
11. Nigeria	2011	MICS	✓	✓	✓	✗	✓	✓
12. Somalia	2006	MICS	✓	✓	✓	✗	✓	✗
13. Timor-Leste	2009	DHS	✓	✓	✓	✓	✓	✓
14. Uganda	2011	DHS	✓	✓	✓	✓	✓	✓
15. Afghanistan								
16. South Sudan				No data available				
17. Papua New Guinea								

* Information on ANC limited to having at least one visit during pregnancy.

Web-appendix 2. Coverage of full immunization and coverage of individual vaccines according to country

Country	UN region	Source	Survey year	Sample	Full immunization coverage			Individual vaccine coverage			
					Fully immunized (N)	Unweighted FIC %	Weighted FIC %	Weighted DPT %	Weighted BCG %	Weighted Measles %	Weighted Polio %
Timor-Leste	East Asia & Pacific	DHS	2009	1805	988	54.8	52.6	66.4	76.7	67.8	56.2
Ethiopia	Eastern & Southern Africa	DHS	2011	1927	565	29.3	24.6	37.0	66.3	55.7	45.1
Somalia	Eastern & Southern Africa	MICS	2006	1096	113	10.3	11.6	14.1	29.7	28.8	37.5
Uganda	Eastern & Southern Africa	DHS	2011	1427	769	53.9	52.5	72.2	93.7	75.8	63.9
Haiti	Latin America & Caribbean	DHS	2012	1370	640	46.7	45.8	62.8	82.7	65.1	59.6
Cameroon	West & Central Africa	DHS	2011	2286	1241	54.3	53.6	68.8	87.1	70.6	70.5
CAR	West & Central Africa	MICS	2010	2015	324	16.1	17.3	32.6	73.6	55.6	45.1
Chad	West & Central Africa	DHS	2004	901	145	16.1	11.4	20.4	40.2	22.8	35.6
Cote dIvoire	West & Central Africa	DHS	2011	1417	716	50.5	50.5	63.8	83.4	64.5	69.2
DR Congo	West & Central Africa	MICS	2010	2318	1122	48.4	49.8	62.6	85.5	72.2	59.9
Guinea	West & Central Africa	DHS	2005	1115	430	38.6	37.4	51.4	79.4	50.2	50.7
Liberia	West & Central Africa	DHS	2007	996	352	35.3	39.1	50.5	77.1	63.0	49.6
Mauritania	West & Central Africa	MICS	2007	1677	651	38.8	35.3	55.5	84.8	74.4	45.4
Nigeria	West & Central Africa	MICS	2011	4755	1458	30.7	33.2	44.8	62.2	55.6	48.6

Web-appendix 3. **Coverage (%) and prevalence ratio of full immunization according to health interventions for 14 selected countries**

Cameroon, 2011

Intervention	Level	N	DPT %	BCG %	Measles %	Polio %	Full immunization coverage FIC %	95%CI	Prevalence ratio	95%CI	p
ANC	0	264	32.4	54.1	38.7	47.5	24.2	16.9, 31.4	1.0	-	<0.001
	1-3	531	62.2	87.6	64.7	68.8	47.5	42.1, 52.9	2.0	1.4, 2.7	
	>3	1359	79.4	94.4	79.6	76.5	62.7	59.3, 66.0	2.6	1.9, 3.5	
SBA	No	703	45.0	69.5	49.5	59.4	32.5	27.3, 37.7	1.0	-	<0.001
	Yes	1467	80.6	95.8	80.8	75.9	64.2	60.9, 67.4	2.0	1.7, 2.3	
VITA	No	948	64.1	84.2	63.5	64.4	47.9	43.4, 52.3	1.0	-	0.001
	Yes	1338	72.0	89.0	75.4	74.7	57.6	53.4, 61.8	1.2	1.1, 1.3	
PNC	No	1292	61.1	82.1	64.7	66.8	46.7	42.4, 51.0	1.0	-	<0.001
	Yes	870	79.8	94.1	78.5	75.7	63.6	59.8, 67.3	1.4	1.2, 1.5	
HVC	No	352	16.5	43.0	30.7	39.1	8.4	5.3, 11.5	1.0	-	<0.001
	Yes	1927	79.5	96.2	78.8	77.1	62.9	59.8, 65.9	7.5	5.2, 10.8	
ITN	No	1965	67.2	86.4	69.2	69.2	51.9	48.3, 55.4	1.0	-	<0.001
	Yes	321	79.8	91.5	80.1	79.3	65.5	59.0, 72.0	1.3	1.1, 1.4	

Central African Republic, 2010

Intervention	Level	N	DPT %	BCG %	Measles %	Polio %	Full immunization coverage: FIC %	95%CI	Prevalence ratio	95%CI	p
ANC	**0**	409	14.0	47.1	35.0	38.6	7.5	3.8, 11.2	1.0	-	<0.001
	**1-3**	702	29.1	74.8	53.5	40.9	14.3	10.7, 17.8	1.9	1.2, 3.2	
	**>3**	728	43.7	86.0	66.5	52.8	23.5	17.9, 29.0	3.1	1.8, 5.5	
**SBA**	**No**	936	21.9	63.8	48.0	39.2	11.7	8.5, 14.9	1.0	-	<0.001
	**Yes**	1042	42.0	82.3	61.6	50.5	21.9	17.5, 26.2	1.9	1.3, 2.6	
**VITA**	**No**	413	18.0	49.6	29.8	35.9	7.3	3.9, 10.7	1.0	-	<0.001
	**Yes**	1602	36.7	80.5	63.0	47.7	20.1	16.6, 23.6	2.8	1.7, 4.5	
**PNC**	**No**	No data available									
	**Yes**										
**HVC**	**No**	389	4.3	38.2	31.3	31.4	1.8	0.1, 3.5	1.0	-	<0.001
	**Yes**	1626	39.1	81.8	61.2	48.2	20.8	17.5, 24.1	11.6	4.5, 30.1	
**ITN**	**No**	1271	33.8	75.5	56.7	45.4	17.9	14.4, 21.3	1.0	-	0.493
	**Yes**	744	30.6	70.6	54	44.6	16.3	12.4, 20.3	0.9	0.7, 1.2	

Coverage (%) and prevalence ratio of full immunization according to health interventions for 14 selected countries

Chad, 2014

							Full immunization coverage				
**Intervention**	**Level**	**N**	**DPT %**	**BCG %**	**Measles %**	**Polio %**	**FIC %**	**95%CI**	**Prevalence ratio**	**95%CI**	**p**
	**0**	390	6.2	19.0	11.3	23.2	2.7	1.0, 4.5	1.0	-	<0.001
**ANC**	**1-3**	246	33.7	61.4	37.7	45.5	23.0	15.9, 30.0	8.5	4.5, 15.8	
	**>3**	236	41.0	71.0	35.1	55.6	20.5	12.6, 28.4	7.5	3.6, 15.7	
**SBA**	**No**	635	16.3	34.4	19.5	33.1	9.5	6.2, 12.8	1.0	-	0.002
	**Yes**	242	40.8	69.5	40.3	47.0	21.1	13.8, 28.4	2.2	1.4, 3.5	
**VITA**	**No**	497	17.0	35.3	17.5	33.0	9.5	6.1, 12.8	1.0	-	0.017
	**Yes**	404	27.0	49.8	33.2	40.7	15.3	10.3, 20.2	1.6	1.1, 2.3	
**PNC**	**No**	774	18.4	37.2	20.8	34.2	10.3	7.0, 13.6	1.0	-	0.020
	**Yes**	102	39.6	69.7	45.2	48.0	22.8	12.7, 32.9	2.2	1.3, 3.7	
**HVC**	**No**	450	7.4	15.5	6.8	26.0	1.7	0.3, 3.1	1.0	-	<0.001
	**Yes**	448	40.7	78.8	47.9	50.2	26.7	19.8, 33.5	16.1	6.6, 39.1	
**ITN**	**No**	No data available									
	**Yes**										

Cote D’Ivoire, 2011

Intervention	Level	N	DPT %	BCG %	Measles %	Polio %	Full immunization coverage: FIC %	95%CI	Prevalence ratio	95%CI	p
ANC	**0**	104	13.9	27.2	29.3	35.7	8.9	3.1, 14.7	1.0	-	<0.001
	**1-3**	654	59.4	83.0	56.9	67.3	45.1	39.4, 50.8	5.1	2.7, 9.6	
	**>3**	612	76.8	92.6	78.2	77.3	63.3	57.9, 68.6	7.1	3.8, 13.5	
**SBA**	**No**	545	44.9	68.7	48.6	58.8	33.1	27.6, 38.6	1.0	-	<0.001
	**Yes**	831	75.5	92.3	74.1	75.8	61.1	56.3, 66.0	1.8	1.6, 2.2	
**VITA**	**No**	473	46.5	70.8	45.9	54.5	32.7	26.6, 38.8	1.0	-	<0.001
	**Yes**	944	73.3	90.2	74.6	77.3	60.3	55.5, 65.1	1.8	1.5, 2.2	
**PNC**	**No**	356	47.1	70.2	51.0	59.3	35.8	28.8, 42.8	1.0	-	<0.001
	**Yes**	1011	70.5	88.4	69.7	73.1	56.3	51.1, 61.4	1.6	1.3, 1.9	
**HVC**	**No**	97	7.1	17.7	22.1	30.5	1.2	0.0, 3.5	NA (1)		
	**Yes**	1319	68.4	88.6	67.9	72.3	54.5	50.1, 58.8			
**ITN**	**No**	800	63.1	84.1	66.1	68.3	50.8	45.3, 56.3	1.0	-	0.861
	**Yes**	617	64.8	82.3	62.2	70.5	50.2	45.0, 55.4	1.0	0.9, 1.1	

NA (1) Insufficient sample size in one of the categories

Coverage (%) and prevalence ratio of full immunization according to health interventions for 14 selected countries

Democratic Republic of the Congo, 2010

Intervention	Level	N	DPT %	BCG %	Measles %	Polio %	Full immunization coverage: FIC %	95%CI	Prevalence ratio	95%CI	p
	**0**	260	30.9	53.0	42.8	36.9	26.6	18.4, 34.8	1.0	-	<0.001
**ANC**	**1-3**	935	64.0	87.7	71.8	61.8	52.3	46.9, 57.8	2.0	1.5, 2.7	
	**>3**	982	68.8	91.6	78.7	64.2	53.2	47.7, 58.7	2.0	1.5, 2.7	
**SBA**	**No**	596	47.6	69.8	59.6	51.7	40.3	33.3, 47.4	1.0	-	<0.001
	**Yes**	1691	68.6	92.0	77.3	63.3	53.7	49.2, 58.2	1.3	1.1, 1.6	
**VITA**	**No**	438	26.6	51.9	29.4	24.3	15.9	11.2, 20.6	1.0	-	<0.001
	**Yes**	1880	69.9	92.2	80.8	67.0	56.6	52.5, 60.8	3.6	2.7, 4.8	
**PNC**	**No**	No data available									
	**Yes**										
**HVC**	**No**	580	14.4	45.5	34.0	19.6	9.3	5.0, 13.6	1.0	-	<0.001
	**Yes**	1738	77.1	97.5	83.7	72.0	62.0	58.1, 66.0	6.7	4.2, 10.6	
**ITN**	**No**	1347	57.4	83.2	69.0	56.6	44.6	40.1, 49.2	1.0	-	<0.001
	**Yes**	971	70.2	88.9	76.9	64.7	57.4	52.2, 62.6	1.3	1.2, 1.4	

Ethiopia, 2011

Intervention	Level	N	DPT %	BCG %	Measles %	Polio %	Full immunization coverage FIC %	95%CI	Prevalence ratio	95%CI	p
ANC	0	991	24.3	53.1	42.7	33.7	14.8	11.0, 18.5	1.0	-	<0.001
	1-3	456	48.8	79.9	70.5	55.5	33.4	27.2, 39.7	2.3	1.7, 3.0	
	>3	398	60.4	88.2	79.5	65.2	42.1	34.6, 49.6	2.9	2.1, 3.8	
SBA	No	1546	33.4	63.4	53.2	42.6	21.1	17.5, 24.7	1.0	-	<0.001
	Yes	305	62.6	86.7	79.8	61.9	48.0	38.8, 57.3	2.3	1.8, 2.9	
VITA	No	820	22.9	53.9	40.5	31.8	14.4	10.5, 18.3	1.0	-	<0.001
	Yes	1107	48.7	76.6	68.3	56.1	32.9	28.3, 37.6	2.3	1.8, 3.0	
PNC	No	1657	34.8	64.6	54.4	43.6	22.4	18.8, 26.0	1.0	-	<0.001
	Yes	191	68.6	89.9	85.4	64.8	53.3	41.1, 65.6	2.4	1.8, 3.1	
HVC	No	726	9.8	37.8	31.8	23.0	5.2	2.3, 8.0	1.0	-	<0.001
	Yes	1197	55.8	85.9	72.1	60.3	37.9	33.3, 42.5	7.3	4.2, 12.7	
ITN	No	No data available									
	Yes										

Coverage (%) and prevalence ratio of full immunization according to health interventions for 14 selected countries

Guinea, 2005

NA 1 Insufficient sample size in one of the categories

							Full immunization coverage				
Intervention	Level	N	DPT %	BCG %	Measles %	Polio %	FIC %	95%CI	Prevalence ratio	95%CI	p
ANC	0	173	9.5	27.4	10.0	13.2	5.1	1.4, 8.9	1.0	-	<0.001
	1-3	315	50.2	80.7	49.9	50.1	37.6	31.1, 44.1	7.3	3.5, 15.3	
	>3	555	64.6	93.4	63.4	62.3	47.2	41.9, 52.5	9.2	4.5, 18.8	
SBA	No	750	43.4	71.9	45.3	45.1	32.8	27.9, 37.8	1.0	-	<0.001
	Yes	339	68.4	95.5	62.6	62.7	48.4	41.4, 55.4	1.5	1.2, 1.8	
VITA	No	331	31.2	57.8	25.6	30.5	17.5	12.4, 22.6	1.0	-	<0.001
	Yes	784	59.6	88.2	60.1	58.8	45.4	40.4, 50.4	2.6	2.0, 3.4	
PNC	No	872	47.8	75.7	46.0	47.5	34.3	29.6, 39.0	1.0	-	<0.001
	Yes	211	63.9	93.1	68.3	62.2	49.9	42.7, 57.1	1.5	1.2, 1.7	
HVC	No	250	9.0	33.6	11.9	15.1	4.2	1.7, 6.6	1.0	-	<0.001
	Yes	864	64.0	93.1	61.6	61.3	47.2	42.8, 51.7	11.3	6.3, 20.2	
ITN	No	1105	51.2	79.3	50.2	50.7	37.5	33.0, 42.0	NA (1)		
	Yes	10	70.8	100.0	55.5	54.0	29.8	0.0, 70.2			

NA (1) Insufficient sample size in one of the categories

Haiti, 2012

Intervention	Level	N	DPT %	BCG %	Measles %	Polio %	Full immunization coverage FIC %	95%CI	Prevalence ratio	95%CI	p
	**0**	124	34.2	54.9	47.0	30.6	21.9	13.5, 30.4	1.0	-	<0.001
**ANC**	**1-3**	306	52.6	79.3	56.0	52.5	37.6	30.0, 45.1	1.7	1.2, 2.5	
	**>3**	870	70.7	88.4	71.1	66.5	51.7	47.5, 55.9	2.4	1.6, 3.4	
**SBA**	**No**	827	57.1	77.5	65.0	55.4	42.2	37.0, 47.4	1.0	-	0.024
	**Yes**	475	72.3	92.0	65.6	66.8	51.1	45.3, 56.8	1.2	1.0, 1.4	
**VITA**	**No**	503	42.1	73.1	37.8	39.1	23.5	18.7, 28.4	1.0	-	<0.001
	**Yes**	867	75.7	88.8	82.3	72.5	59.7	55.5, 63.9	2.5	2.1, 3.1	
**PNC**	**No**	923	59.5	79.3	64.5	58.1	44.0	39.3, 48.8	1.0	-	0.220
	**Yes**	374	70.6	91.9	66.8	63.2	48.8	42.5, 55.1	1.1	0.9, 1.3	
**HVC**	**No**	149	13.8	45.0	30.8	11.4	9.2	3.3, 15.0	1.0	-	<0.001
	**Yes**	1219	69.1	87.7	69.6	65.8	50.5	46.7, 54.3	5.5	2.9, 10.3	
**ITN**	**No**	1156	61.1	81.1	63.7	58.1	43.4	39.1, 47.6	1.0	-	0.002
	**Yes**	214	72.1	91.9	73.1	67.8	59.0	50.7, 67.3	1.4	1.1, 1.6	

Coverage (%) and prevalence ratio of full immunization according to health interventions for 14 selected countries

Liberia, 2007

							Full immunization coverage				
**Intervention**	**Level**	**N**	**DPT %**	**BCG %**	**Measles %**	**Polio %**	**FIC %**	**95%CI**	**Prevalence ratio**	**95%CI**	**p**
	**0**	36	10.1	41.2	30.3	11.1	9.3	0.0, 22.6	1.0	-	<0.001
**ANC**	**1-3**	199	26.4	52.6	39.2	33.2	22.6	13.9, 31.3	2.4	0.6, 9.3	
	**>3**	604	58.7	86.4	72.0	55.1	44.9	39.6, 50.2	4.8	1.3, 18.4	
**SBA**	**No**	478	37.6	63.9	51.3	40.0	28.8	21.9, 35.7	1.0	-	<0.001
	**Yes**	475	63.7	91.2	74.6	59.2	49.3	43.5, 55.0	1.7	1.3, 2.2	
**VITA**	**No**	483	40.5	68.2	51.5	40.0	29.9	23.3, 36.6	1.0	-	<0.001
	**Yes**	513	61.2	86.6	75.4	59.9	49.0	42.2, 55.7	1.6	1.3, 2.1	
**PNC**	**No**	372	33.9	61.4	48.4	36.0	25.7	18.7, 32.7	1.0	-	<0.001
	**Yes**	574	60.7	87.3	71.7	57.9	47.0	41.5, 52.6	1.8	1.4, 2.4	
**HVC**	**No**	239	7.9	25.2	25.8	13.9	3.4	0.5, 6.3	1.0	-	<0.001
	**Yes**	754	62.9	92.2	73.9	60.0	49.5	44.6, 54.3	14.6	6.3, 34.0	
**ITN**	**No**	No data available									
	**Yes**										

Mauritania, 2007

Intervention	Level	N	DPT %	BCG %	Measles %	Polio %	Full immunization coverage FIC %	95%CI	Prevalence ratio	95%CI	p
	**0**	480	50.2	75.9	67.3	45.9	37.2	32.0, 42.4	1.0	-	0.459
**ANC**	**1+**	1128	58.9	89.3	77.9	45.3	35.1	31.4, 38.7	0.9	0.8, 1.1	
**SBA**	**No**	733	53.9	79.2	70.3	49.1	39.5	35.1, 43.9	1.0	-	0.016
	**Yes**	875	58.2	90.2	78.2	42.6	32.6	28.5, 36.7	0.8	0.7, 1.0	
**VITA**	**No**	671	46.7	81.8	68.5	38.5	27.4	23.4, 31.5	1.0	-	<0.001
	**Yes**	1006	62.5	87.2	79.0	50.7	41.5	37.7, 45.4	1.5	1.3, 1.8	
**PNC**	**No**	No data available									
	**Yes**										
**HVC**	**No**	222	20.1	44.3	35.1	10.7	4.5	1.8, 7.2	1.0	-	<0.001
	**Yes**	1455	61.4	91.5	80.9	51.1	40.4	36.8, 44.0	9.0	4.9, 16.5	
**ITN**	**No**	No data available									
	**Yes**										

Coverage (%) and prevalence ratio of full immunization according to health interventions for 14 selected countries

Nigeria, 2007

Intervention	Level	N	DPT %	BCG %	Measles %	Polio %	Full immunization coverage: FIC %	95% CI	Prevalence ratio	95%CI	p
ANC	0	1427	10.3	23.6	21.0	27.2	7.0	5.0, 8.9	1.0	-	<0.001
	1-3	483	39.8	59.3	54.8	48.7	29.6	24.1, 35.1	4.3	3.1, 5.9	
	>3	2303	64.5	83.3	73.6	61.3	49.1	45.5, 52.7	7.1	5.3, 9.4	
SBA	No	2733	22.9	40.0	35.4	37.1	16.7	14.5, 18.8	1.0	-	<0.001
	Yes	1923	69.6	87.4	78.6	62.0	52.3	48.7, 56.0	3.1	2.7, 3.6	
VITA	No	1755	22.9	35.5	29.8	31.4	17.3	14.1, 20.5	1.0	-	<0.001
	Yes	3000	56.9	76.9	69.9	58.1	42.0	38.9, 45.1	2.4	2.0, 2.9	
PNC	No	No data available									
	Yes										
HVC	No	2184	6.4	21.7	20.7	25.5	3.1	2.1, 4.0	1.0	-	<0.001
	Yes	2571	73.6	92.4	81.8	65.9	55.8	52.8, 58.7	18.3	13.2, 25.3	
ITN	No	3869	44.4	61.1	54.4	48.1	32.8	29.9, 35.7	1.0	-	0.454
	Yes	886	46.9	67.0	61.2	51.1	35.0	29.9, 40.0	1.1	0.9, 1.3	

Somalia, 2006

NA 1 Insufficient sample size in one of the categories

Intervention	Level	N	DPT %	BCG %	Measles %	Polio %	Full immunization coverage FIC %	95%CI	Prevalence ratio	95%CI	p
ANC	0	646	9.4	21.1	22.7	31.4	8.4	5.5, 11.4	1.0	-	<0.001
	1-3	272	24.3	47.5	41.7	51.1	19.7	13.4, 26.0	2.3	1.6, 3.5	
	>3	12	42.4	66.1	58.2	41.6	31.1	0.0, 66.9	NA (1)	NA (1)	
SBA	No	690	9.2	22.1	23.2	30.1	7.5	4.8, 10.3	1.0	-	<0.001
	Yes	367	24.3	44.9	41.1	52.5	20.9	14.9, 26.8	2.8	1.9, 4.0	
VITA	No	855	11.2	25.1	24.2	34.1	9.2	6.3, 12.1	1.0	-	0.002
	Yes	241	23.6	45.2	44.2	49.0	19.7	12.8, 26.6	2.1	1.4, 3.1	
PNC	No	No data available									
	Yes										
HVC	No	802	2.5	11.6	15.2	27.3	1.3	0.5, 2.1	1.0	-	<0.001
	Yes	294	43.8	76.3	63.7	63.8	38.1	30.2, 46.0	29.3	15.7, 54.6	
ITN	No	No data available									
	Yes										

NA (1) Insufficient sample size in one of the categories

Coverage (%) and prevalence ratio of full immunization according to health interventions for 14 selected countries

Uganda, 2011

Intervention	Level	N	DPT %	BCG %	Measles %	Polio %	Full immunization coverage				
							FIC %	95%CI	Prevalence ratio	95%CI	p
ANC	0	43	41.6	68.7	50.8	42.9	33.3	12.2, 54.3	1.0	-	0.029
	1-3	594	69.6	94.3	73.4	63.0	50.7	45.9, 55.5	1.5	0.8, 2.8	
	>3	665	77.1	95.6	79.7	67.7	57.2	52.2, 62.2	1.7	0.9, 3.1	
SBA	No	514	67.6	90.4	72.0	61.0	50.2	45.0, 55.5	1.0	-	0.139
	Yes	811	75.2	96.1	78.2	66.5	54.9	50.5, 59.4	1.1	1.0, 1.2	
VITA	No	508	57.6	87.1	61.1	49.4	35.0	30.2, 39.7	1.0	-	<0.001
	Yes	919	80.5	97.4	84.0	72.1	62.4	58.3, 66.6	1.8	1.5, 2.1	
PNC	No	848	68.8	92.0	73.0	62.4	50.0	45.5, 54.4	1.0	-	0.011
	Yes	470	78.6	97.2	81.0	67.8	58.6	53.1, 64.1	1.2	1.0, 1.3	
HVC	No	170	42.4	71.1	50.5	20.3	10.6	4.7, 16.5	1.0	-	<0.001
	Yes	1256	76.0	96.6	79.0	69.5	57.8	54.2, 61.5	5.5	3.2, 9.5	
ITN	No	739	65.9	92.0	72.1	57.9	47.1	42.7, 51.5	1.0	-	<0.001
	Yes	688	78.9	95.5	79.6	70.2	58.3	53.3, 63.2	1.2	1.1, 1.4	

Timor-Leste, 2009

Intervention	Level	N	DPT %	BCG %	Measles %	Polio %	Full immunization coverage: FIC %	95%CI	Prevalence ratio	95%CI	p
	**0**	200	23.2	33.1	25.9	23.6	20.1	13.8, 26.5	1.0	-	<0.001
**ANC**	**1-3**	511	65.8	79.3	68.1	56.3	52.5	47.1, 57.9	2.6	1.9, 3.6	
	**>3**	953	76.2	85.1	76.6	63.6	60.0	55.6, 64.4	3.0	2.2, 4.1	
**SBA**	**No**	1185	60.9	70.9	62.2	52.8	48.8	44.7, 52.9	1.0	-	<0.001
	**Yes**	490	79.0	90.3	80.3	64.5	61.5	55.8, 67.3	1.3	1.1, 1.4	
**VITA**	**No**	757	49.1	58.8	48.0	39.6	34.9	30.3, 39.5	1.0	-	<0.001
	**Yes**	1046	81.3	92.1	84.7	70.4	67.7	63.6, 71.8	1.9	1.7, 2.2	
**PNC**	**No**	1315	62.2	72.1	63.7	53.8	50.0	46.0, 54.0	1.0	-	<0.001
	**Yes**	355	81.1	93.8	81.8	65.7	62.5	56.1, 68.9	1.3	1.1, 1.4	
**HVC**	**No**	542	27.5	37.9	32.7	14.7	13.5	9.9, 17.1	1.0	-	<0.001
	**Yes**	1261	84.5	94.7	84.1	75.4	70.7	67.0, 74.4	5.2	4.0, 6.8	
**ITN**	**No**	1010	58.2	69.3	60.1	50.1	46.3	42.1, 50.4	1.0	-	<0.001
	**Yes**	793	77.1	86.4	77.8	64.0	60.8	55.7, 65.8	1.3	1.2, 1.5	
